# High‐Entropy Engineering and External Magnetic Field Modulation Synergistically Enhance the H_2_S Sensing Performance of Rare‐Earth Perovskite Ferrites

**DOI:** 10.1002/advs.76945

**Published:** 2026-08-05

**Authors:** Yunfei Wang, Min Zhang, Rui Li, Xiaolong Yao, Zhaofeng Wu, Fengdong Qu, Shan Qiu, Zhenjiang Li

**Affiliations:** ^1^ School of Physics Science and Technology Xinjiang University Urumqi China; ^2^ School of Materials Science and Engineering Xinjiang University Urumqi China; ^3^ School of Microelectronics Northwestern Polytechnical University Xi'an China

**Keywords:** gas sensors, high‐entropy perovskite oxides, lattice distortions, magnetic fields, rare‐earth ferrites

## Abstract

High‐performance gas sensors with extreme sensitivity and rapid response kinetics are fundamental to sub‐ppm H_2_S detection, which is crucial for environmental monitoring and industrial safety. However, most conventional rare‐earth ferrites have relatively sluggish responses at low concentrations because of thermodynamic and kinetic constraints and inherently high surface reaction barriers. Herein, a high‐entropy perovskite oxide with the composition (Gd_0.2_Tb_0.2_Dy_0.2_Ho_0.2_Er_0.2_)FeO_3_ was designed and synthesized using a wet‐chemical method. The incorporation of multiple principal elements induced strong local lattice distortion and generated abundant chemically heterogeneous active sites. Furthermore, the application of an external magnetic field significantly optimized electron transfer pathways and accelerated the surface redox reaction kinetics. Our results reveal that the synergistic coupling between high‐entropy‐induced structural distortion and weak magnetic field modulation breaks the traditional response‐recovery trade‐off, yielding a 6‐fold sensitivity enhancement over conventional counterparts, an ultrafast 1.72 s recovery time, and an H_2_S detection limit of 0.5 ppm. This strategy, which integrates local coordination reconstruction with spin‐state intervention, is expected to be widely applicable to the development of next‐generation intelligent sensing systems.

## Graphical abstract

A novel high‐entropy perovskite oxide, (Gd_0.2_Tb_0.2_Dy_0.2_Ho_0.2_Er_0.2_)FeO_3_, was successfully synthesized for advanced hydrogen sulfide gas detection. By synergistically coupling high‐entropy‐induced local lattice distortion with weak magnetic field modulation, the tailored material generates abundant active sites. This innovative dual strategy dramatically amplifies the sensing signal and accelerates detection speeds, providing a highly effective approach for next‐generation sensor design.

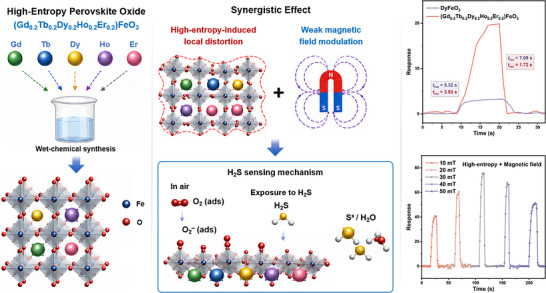

## 1 Introduction

Hydrogen sulfide (H_2_S), a highly toxic ubiquitous pollutant, can cause irreversible health effects even at sub‐ppm levels, necessitating the urgent development of gas sensors with both ultrahigh sensitivity and ultrafast response kinetics [1, 2, 3]. Among numerous candidate systems, considerable attention has been directed toward rare‐earth ferrites (*R*FeO_3_), a class of strongly correlated perovskite semiconductors [4, 5]. The similar ionic radii and 4*f* electronic configurations of the A‐site lanthanide ions confer remarkable electronic structure tunability upon these materials [6], whereas the B‐site Fe 3*d* states and the strong *p*–*d* orbital hybridization while the B‐site Fe 3*d* states and strong *p*–*d* orbital hybridization within the Fe─O framework provide abundant surface active sites and excellent catalytic activity toward redox reactions [7, 8, 9]. Nevertheless, conventional pristine *R*FeO_3_ materials are limited by relatively high surface reaction barriers, resulting in intrinsically sluggish responses at low analyte concentrations. To overcome this limitation, employing the size effect of A‐site ions to induce local lattice distortion has emerged as a key strategy for reshaping the electronic structure of the electronic structures of these materials. A‐site substitution directly modulates the lattice tolerance factor, driving the distortion of the FeO_6_ octahedra, and breaking local symmetry [10, 11] Such pronounced lattice distortion reconstructs the coordination environment of the active Fe centers and modulates their local density of states [7]. This not only significantly enhances the surface coverage of highly reactive chemisorbed oxygen species (e.g., O_2_^−^, O^−^) but also strengthens the direct hybridization and resonance between surface Fe 3*d* orbitals and the frontier orbitals of H_2_S [12]. This intrinsic activation mechanism, based on local lattice distortion and orbital reconstruction, effectively lowers the interfacial charge transfer barrier, thereby providing a physical basis for achieving ultrafast catalytic reactions.

Although conventional single‐element substitution can induce the aforementioned structural evolutions, further intensification of lattice perturbations is required to maximize the activation of distortion‐driven electronic reconstruction. On this basis, the emerging concept of high‐entropy oxides (HEOs) offers a revolutionary materials design strategy to transcend the limitations of traditional doping and develop sensing materials with exceptional performance [13, 14, 15, 16, 17]. Unlike the delicate tuning approach using only a limited number of elements, the A‐site high‐entropy strategy employs five or more rare‐earth elements, each possessing distinct ionic radii, electronegativities, and outermost electronic configurations, to jointly occupy the A‐site in near‐equimolar ratios [18]. Thermodynamically, the high configurational entropy (Δ*S*_conf_) dominates the Gibbs free energy (Δ *G*_mix_ =  Δ*H*_mix_ − *T*Δ*S*_mix_), effectively overcoming the positive mixing enthalpy (Δ*H*_mix_) associated with multi‐element mixing and stabilizing a single‐phase solid solution [19]. From a crystallographic perspective, this atomic‐scale compositional disorder induces substantial lattice microstrain and local structural distortions [20]. The resulting heterogeneous stress field renders the coordination environment of surface atoms extremely complex, thereby giving rise to a broadened surface density of states (DOS). More importantly, the synergistic “cocktail effect” arising from multiple principal elements not only creates abundant sub‐nanometer‐scale multi‐element catalytic active sites on the material surface but also reshapes the charge carrier scattering and transport pathways. This endows the material with unprecedented advantages in the kinetics of gas molecule adsorption and desorption [21].

Beyond the fundamental reconstruction of the material's intrinsic structure, external magnetic field modulation, as a non‐contact spin regulation method, is emerging as a cutting‐edge approach to overcome critical performance bottlenecks in sensing [22, 23]. Owing to both the intrinsic magnetic properties of *R*FeO_3_‐based materials, including the antiferromagnetic‐to‐weak‐ferromagnetic phase transition [5, 24, 25], and the paramagnetic nature of O_2_ molecules [26], the introduction of an external magnetic field profoundly modulates the spin‐dependent reaction kinetics at the gas‐solid interface. Mechanistically, the external magnetic field induces, via the Zeeman effect or spin polarization, a reconfiguration of unpaired electrons on the surface Fe ions. This alleviates spin restrictions during O_2_ adsorption and consequently lowers the activation energy of the surface reaction. Concurrently, through the Lorentz force and spin‐dependent scattering mechanisms, the magnetic field dynamically modulates both the width of the electron depletion layer and the carrier mobility within the mesoporous semiconductor network, thereby achieving a substantial amplification of the electrical signal corresponding to the target gas [23].

Herein, we propose a synergistic strategy integrating high‐entropy‐induced lattice distortions and external magnetic field modulation to achieve atomic‐scale control over surface catalytic kinetics. Based on the intrinsic spin‐modulated properties of *R*FeO_3_ systems, a five‐component high‐entropy ferrite, (Gd_0.2_Tb_0.2_Dy_0.2_Ho_0.2_Er_0.2_)FeO_3_, was designed. While the extremely high configurational entropy stabilizes a single orthorhombic phase, multi‐principal‐element size mismatches induce substantial local structural distortions. Synchrotron pair distribution function (PDF) and density functional theory (DFT) analyses confirm that this broken local symmetry broadens the atomic pair distance distribution and promotes strong overlap between Fe 3*d* and S 3*p* orbitals, substantially enhancing the chemisorption energy of H_2_S at Fe active centers. Consequently, the increased abundance of adsorbed oxygen species resulting from structural distortions, coupled with the magnetic‐field‐modulated carrier transport, markedly accelerates the surface redox kinetics. Compared to its pristine counterpart, this high‐entropy sensor exhibits a sixfold enhancement in its response to 3 ppm H_2_S. Notably, upon application of an external magnetic field, the sensor demonstrates pronounced kinetic enhancements. Specifically, its response signal increases by more than fourfold, thereby lowering the limit of detection to 0.5 ppm. This outstanding performance not only overcomes the kinetic limitations of conventional rare‐earth ferrites but also provides an innovative design paradigm for developing high‐performance sensing materials under multi‐field coupling conditions.

## 2 Results and Discussion

Perovskite‐type GdFeO_3_ (GFO), TbFeO_3_ (TFO), DyFeO_3_ (DFO), HoFeO_3_ (HFO), ErFeO_3_ (EFO), and high‐entropy perovskite (Gd_0.2_Tb_0.2_Dy_0.2_Ho_0.2_Er_0.2_)FeO_3_ (GTDHEFO) samples were synthesized using a sol–gel method. Tolerance factor calculations were performed for each component [27] (Text S1), confirming that all compositions satisfied the geometric requirements for the formation of orthorhombic perovskite phases. This provided a theoretical foundation for the successful synthesis of the high‐entropy sample. To quantitatively analyze the concentrations of nearly all metallic elements present in the high‐entropy sample, inductively coupled plasma optical emission spectrometry (ICP‐OES) was performed (Table S1). The actual composition of the GTDHEFO sample was calculated to be (Gd_0.21_Tb_0.19_Dy_0.21_Ho_0.19_Er_0.20_)FeO_3_, which is in excellent agreement with the designed stoichiometry.

**FIGURE 1:**
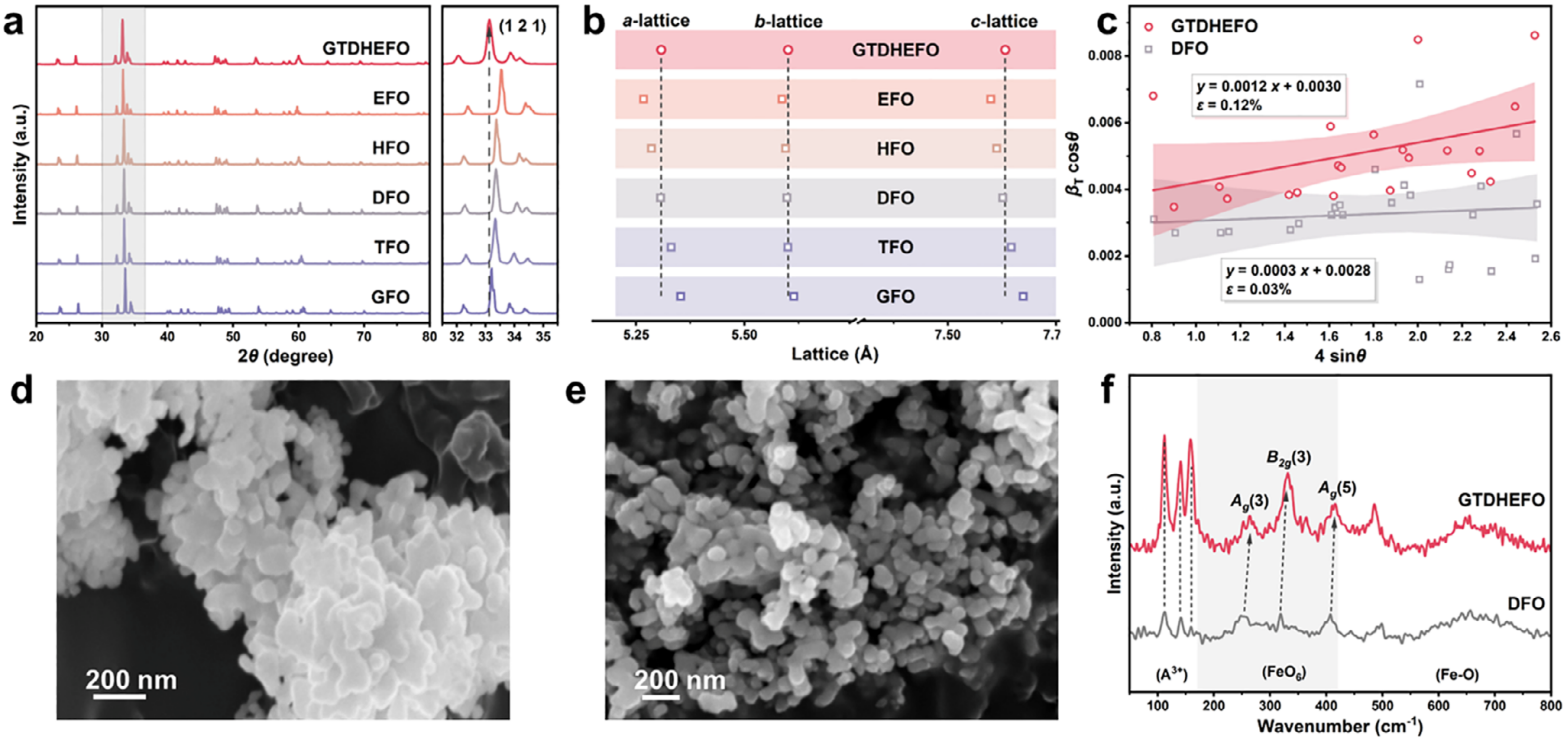
Structural characterization of the pristine and high‐entropy orthoferrites. (a) XRD patterns of the GFO, TFO, DFO, HFO, EFO, and GTDHEFO particles. The right panel shows an enlarged normalized overlay of the (121) diffraction peaks of GTDHEFO and DFO. (b) Evolution of lattice parameters (*a*, *b*, *c*) across the constituent oxides compared to the high‐entropy GTDHEFO (red circles). (c) Williamson‐Hall plots for GTDHEFO and DFO, illustrating the linear fit used to calculate lattice microstrain. (d, e) SEM images of DFO and GTDHEFO. (f) Comparative Raman spectra of DFO and GTDHEFO. The dashed lines highlight the blueshift of the *A*_g_(3) and *A*_g_(5) modes in the high‐entropy sample, reflecting local lattice distortion.

Figure 1a presents the X‐ray diffraction (XRD) patterns of a series of pristine rare‐earth ferrites (GFO–EFO) and a high‐entropy GTDHEFO sample. The results demonstrate that all samples crystallize into a single orthorhombic perovskite phase (space group *Pnma*), with no impurity phases detected. As the atomic number of the rare‐earth ions increases, the characteristic diffraction peaks (e.g., the (121) reflection) systematically shift toward higher angles, a trend attributed to the reduction in ionic radius caused by the lanthanide contraction effect. The Rietveld refinements of the XRD patterns (Figure S1 and Table S2) further quantified this structural evolution, confirming that the lattice constants *a*, *b*, and *c* decrease monotonically with increasing atomic number.

Notably, the refined lattice parameters of GTDHEFO lie between those of its constituent end‐members and remain very close to those of DFO (Figure 1b and Table S2). The slightly broader diffraction peaks of GTDHEFO arise from its enhanced lattice microstrain. This observation indicates a homogeneous solid solution of the five rare‐earth cations within the A‐site sublattice. To obtain further information, a typical Williamson‐Hall analysis [28] was performed (Figure 1c). The calculation details are provided in Text S2. Scanning electron microscopy (SEM) observations reveal that both DFO and GTDHEFO exhibit a sub‐spherical particle morphology (Figure 1d,e). To quantitatively evaluate the particle size, statistical analysis was performed based on the microscopy images (Figure S2). The average particle sizes of DFO and GTDHEFO are 74.10 ± 1.29 nm and 70.83 ± 0.70 nm, respectively. The highly comparable particle size distributions indicate that the broader XRD peaks of GTDHEFO are not primarily caused by a particle‐size effect. The calculated lattice microstrain of GTDHEFO reached 0.12%, approximately four times that of the structurally analogous reference compound DFO (0.03%). This pronounced strain enhancement is ascribed to the inherent “cocktail effect” in high‐entropy materials, whereby multiple cations with different radii, masses, and electronic configurations occupy the same crystallographic site, inducing severe local atomic‐scale lattice distortions and consequently yielding a substantial increase in the overall lattice microstrain. Batch‐to‐batch reproducibility was further evaluated using three independently synthesized GTDHEFO samples prepared under identical conditions (Figure S3). All three batches show the same perovskite phase without detectable impurities and similar sub‐spherical morphology, with average particle sizes of 70.60, 73.88, and 81.66 nm, confirming the good reproducibility of the synthesis process.

To uncover the structural origin of the observed lattice microstrain, the effective coordination number (ECoN) [29] and lattice distortion parameter (*ϵ*) [30] were calculated based on Rietveld refinement data (Texts S3 and S4 and Tables S3 and S4). In the orthorhombic *Pnma* structure, the cooperative tilting of the B‐site oxygen octahedra induces severe distortion of the A‐site coordination polyhedron, with bond lengths to some oxygen atoms being significantly elongated. Consequently, the theoretical ECoN of the A‐site cation is typically reduced to approximately eight. Notably, while the rigid FeO_6_ octahedra maintain a consistent ECoN (∼5.98) in both samples, the A‐site ECoN in GTDHEFO (7.02) is substantially lower than that in DFO (7.17). This reduction in the effective coordination number of the A‐site environment is attributed to the substantial size mismatch among the multiple principal cations, which broadens the distribution of *R*─O bond lengths. The markedly elongated bonds contribute less to the weighted coordination number, thereby suppressing the ECoN value. Concurrently, GTDHEFO exhibits a more pronounced geometric distortion, as reflected by an increased *ɛ* value. The combination of a reduced A‐site ECoN and an increased lattice distortion parameter robustly confirms the presence of pronounced atomic‐scale stress and distortion, which serve as the microscopic origin of the lattice microstrain observed in the Williamson–Hall analysis.

As a complement to the average structural information obtained from XRD, Raman spectroscopy further probed the local lattice dynamics (Figure 1f). The acquired spectra are divided into three regions: low‐frequency modes (< 200 cm^−1^) associated with the translation of A‐site cations (*R* ^3+^), intermediate‐frequency modes (200–450 cm^−1^) corresponding to tilt modes of the FeO_6_ octahedra, and high‐frequency modes (> 450 cm^−1^) attributed to Fe─O stretching vibrations [31, 32]. Whereas the low‐frequency modes of DFO and GTDHEFO exhibit negligible differences, pronounced blueshifts (i.e., toward higher wavenumbers) of the *A*_g_(3) and *A*_g_(5) modes are observed for GTDHEFO in the intermediate‐frequency region. These two modes correspond to the in‐phase rotations of FeO_6_ octahedra along the [010]_pc_ and [101]_pc_ axes, respectively [33, 34]. According to the linear relationship established by Weber et al., such blueshifts imply an increase in the octahedral tilt angle (*Φ*), indicating a more pronounced perovskite structural distortion [33]. This spectroscopic evidence strongly supports the aforementioned microstrain analysis, confirming that local distortions induced by the multi‐principal A‐site elements not only alter the static structure but also profoundly influence the rotational dynamics of the FeO_6_ framework. Thus, the severe lattice distortion characteristic of the high‑entropy system is unambiguously verified.

**FIGURE 2:**
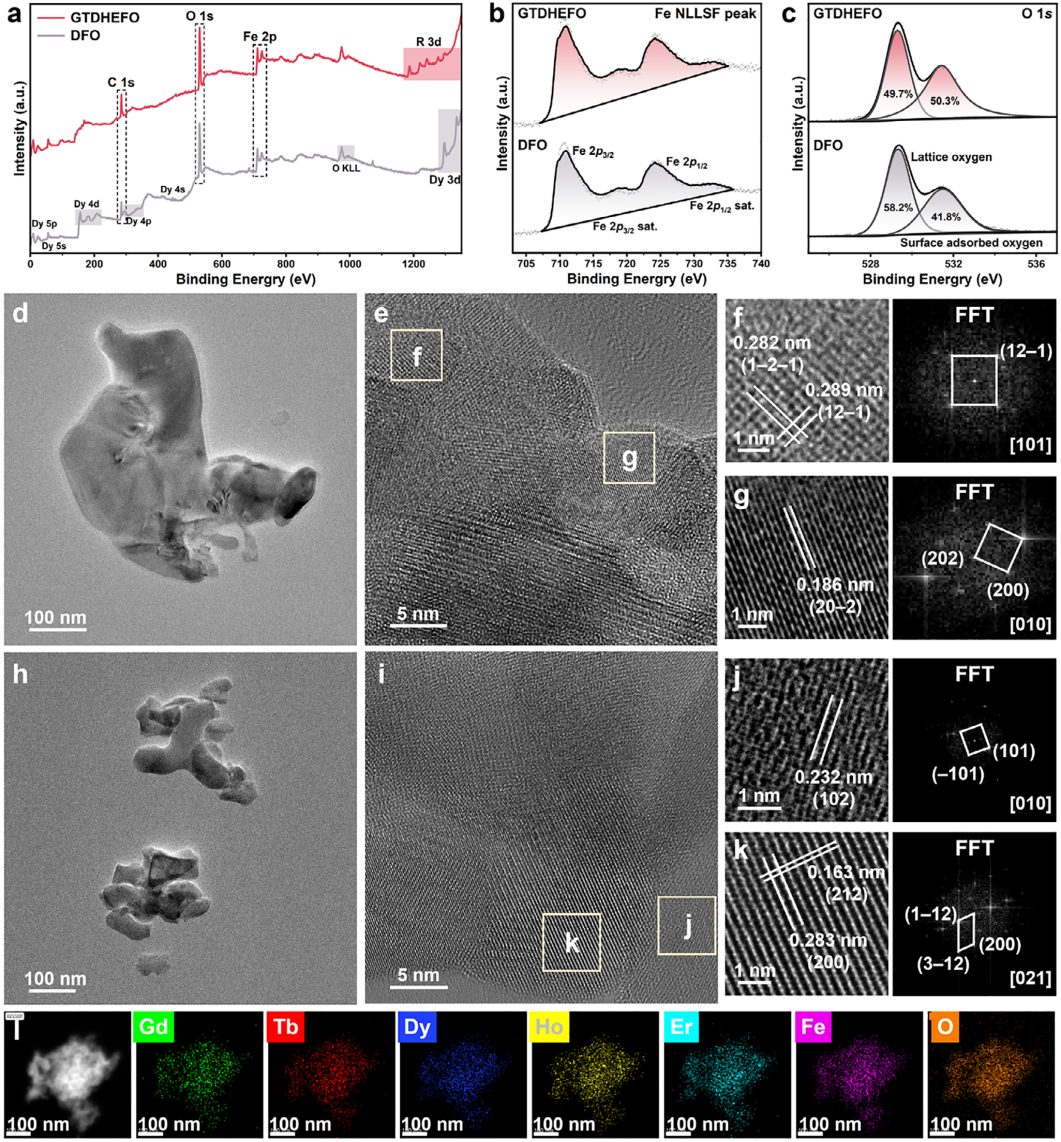
Chemical and local structural analysis. (a) XPS full survey spectra for the pristine and high‐entropy particles. (b) High‐resolution Fe 2*p* XPS spectra for DFO and GTDHEFO fitted using the NLLSF method, showing the characteristic Fe^3+^ satellite peaks. (c) Deconvoluted high‐resolution O 1*s* spectra, illustrating the proportions of lattice oxygen (O_L_) and surface adsorbed oxygen (O_A_). (d, h) TEM images showing the morphology of DFO and GTDHEFO particles. (e, i) HRTEM images displaying high crystallinity. (f, g) Enlarged HRTEM areas of DFO with FFT insets, indexing the (1–21), (12–1), and (20–2) planes. (j, k) Enlarged HRTEM areas of GTDHEFO with FFT insets, identifying the (102), (212), and (200) planes along the [010] zone axis. (l) STEM‐EDS elemental mapping of GTDHEFO, confirming the homogeneous distribution of all constituent elements.

To thoroughly investigate the surface chemical states and elemental composition of the entropy‐stabilized perovskite oxide, X‐ray photoelectron spectroscopy (XPS) analysis was conducted. The survey spectrum (Figure 2a) confirms the successful synthesis of the high‐entropy phase. Characteristic core‐level peaks corresponding to Gd, Tb, Dy, Ho, and Er are simultaneously observed in the spectrum of GTDHEFO, verifying the successful coexistence of the five distinct rare‐earth cations at the surface level. In addition to the XPS survey spectrum, high‐resolution XPS in the 130–180 eV region was performed to further identify the rare‐earth elements in GTDHEFO (Figure S4), where the deconvoluted components can be assigned to Gd 4*d*, Tb 4*d*, Dy 4*d*, Ho 4*d*, and Er 4*d*. To determine the valence states of the B‐site cations, a nonlinear least‐squares fitting (NLLSF) analysis was performed on the high‐resolution Fe 2*p* spectra of DFO and GTDHEFO (Figure 2b) [35]. Both samples exhibit a typical spin‐orbit‐split doublet, comprising Fe 2*p*_3/2_ and Fe 2*p*_1/2_ [36, 37]. Importantly, a distinct satellite peak is observed approximately 8 eV above the main Fe 2*p*_3/2_ peak. This feature is characteristic of the Fe^3+^ oxidation state, thereby ruling out the presence of metallic Fe or significant Fe^2+^. Notably, the spectral profile of the high‐entropy GTDHEFO is almost identical to that of the pristine DFO, indicating that, despite the severe A‐site disorder, the fundamental Fe^3+^ valence state is well preserved. Further analysis of surface oxygen species was performed through peak fitting of the O 1*s* spectra (Figure 2c). Given that electron paramagnetic resonance (EPR) reveals an extremely low bulk oxygen vacancy concentration (Figure S5), the O 1*s* peaks were deconvoluted into two components: lattice oxygen (O_L_, metal‐oxygen bonds) and surface‐adsorbed oxygen (O_A_, e.g., O_2_^−^, and O^−^). Quantitative fitting shows that the O_L_ and O_A_ fractions are 58.2% and 41.8% for DFO, respectively, whereas the corresponding values for GTDHEFO are 49.7% and 50.3%. The markedly higher O_A_ fraction in GTDHEFO indicates enhanced surface oxygen adsorption, which is beneficial for gas sensing because it provides more active oxygen species for surface redox reactions.

To further support the surface oxygen analysis, O_2_ temperature‐programmed desorption (O_2_‐TPD) and mass spectrometry‐monitored O_2_‐H_2_ temperature‐programmed desorption/reduction (O_2_‐H_2_‐TPD‐MS) were performed to assess the oxygen adsorption/activation capacity and reduction reactivity of DFO and GTDHEFO (Figure S6). Compared with DFO, which exhibits a weak O_2_ desorption peak at 373.9 K with a desorption amount of 0.00359 mmol g^−1^, GTDHEFO shows a stronger low‐temperature O_2_ desorption peak at 361.9 K with a much larger desorption amount of 0.06509 mmol g^−1^. In addition, GTDHEFO exhibits stronger *m*/*z* = 18, 44, and 16 signals under the O_2_‐H_2_‐TPD‐MS conditions. These results indicate that high‐entropy engineering increases the abundance and reactivity of labile surface oxygen species, which is favorable for H_2_S‐related surface redox reactions and interfacial charge transfer.

To investigate the microstructure and crystallographic features of the samples, transmission electron microscopy (TEM), and high‐resolution transmission electron microscopy (HRTEM) were employed. As shown in Figure 2d,h, DFO and GTDHEFO exhibit dispersed particles with sub‐spherical morphologies, and their sizes are consistent with the SEM observations (Figure 1d,e), indicating that the high‐entropy configuration does not significantly alter the grain growth habit. The HRTEM images (Figure 2e,i) reveal clear and continuous lattice fringes extending to the edges, confirming the high crystallinity of the samples and the absence of amorphous phases. For DFO, the interplanar spacings of 0.282, 0.289, and 0.186 nm (Figure 2f,g) correspond to the (1–21), (12–1), and (20–2) planes of the orthorhombic *Pnma* structure, respectively. Similarly, GTDHEFO exhibits clear fringes with spacings of 0.232, 0.163, and 0.283 nm, which are assigned to the (102), (212), and (200) planes, respectively (Figure 2j,k). Notably, the fast Fourier transform (FFT) diffraction spots indicate that the electron beam was incident parallel to the [010] zone axis. This crystallographic orientation implies that the particles preferentially exposed the (010) plane, which results from the thermodynamic drive to minimize the total surface energy, as the (010) plane is a stable low‐energy terminating surface in the orthorhombic *Pnma* structure. This finding is consistent with previously reported experimental observations [38, 39, 40]. In addition, energy‐dispersive X‐ray spectroscopy (EDS) elemental mapping was employed to assess the chemical homogeneity of the high‐entropy particles (Figure 2l). The maps reveal that all constituent cations (Gd, Tb, Dy, Ho, Er, and Fe) and oxygen were homogeneously distributed within the particles, without any elemental segregation or secondary phase precipitation, confirming the successful synthesis of a homogeneous single‐phase high‐entropy solid solution.

**FIGURE 3:**
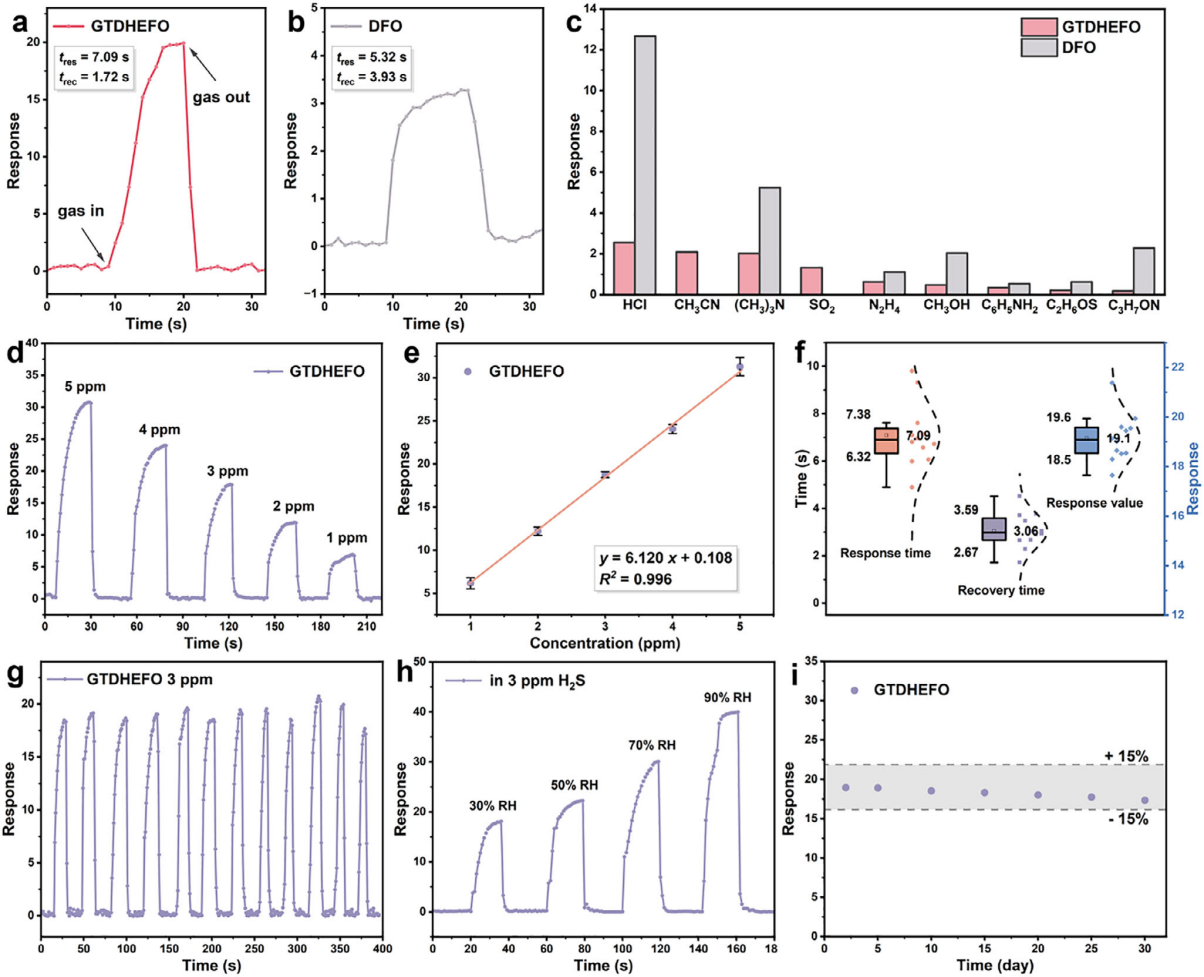
H_2_S gas sensing performance. (a, b) Single‐cycle transient response curves of GTDHEFO and DFO toward 3 ppm H_2_S, showing the response and recovery time. (c) Selectivity histogram comparing the response of GTDHEFO and DFO to various interfering gases (500 ppm). (d) Dynamic response‐recovery curves of GTDHEFO to varying H_2_S concentrations (1–5 ppm). (e) Linear fitting of the response value vs. H_2_S concentration. (f) Statistical box plots summarizing the distributions of response values, response times, and recovery times. (g) Repeatability test of GTDHEFO over 12 consecutive cycles at 3 ppm. (h) Dynamic response–recovery curves of GTDHEFO toward 3 ppm H_2_S under different RH conditions. (i) Long‐term stability assessment of the GTDHEFO sensor exposed to 3 ppm H_2_S over a 30‐day period.

To assess the potential of entropy‐stabilized perovskites as high‐performance chemiresistive sensors, the most critical parameter, namely sensitivity, was first investigated. As shown in Figure 3a, the GTDHEFO sensor exhibits a response value as high as 18.74 upon exposure to only 3 ppm of H_2_S, which is approximately six times the response of the pristine DFO sensor, highlighting the significant enhancement in sensitivity achieved through the high‐entropy design. This performance improvement is attributed to the complex A‐site chemical environment introduced by the “cocktail effect” in GTDHEFO. The incorporation of cations with smaller ionic radii increases the local charge density, thereby generating stronger Lewis acid sites [41, 42]. According to relevant DFT and mechanistic studies, these sites can more effectively attract the lone‐pair electrons of the sulfur atom in H_2_S, exerting a multi‐element synergistic effect to promote the charge transfer process [43], thereby greatly enhancing the response signal [44]. From a structural perspective, Brunauer‐Emmett‐Teller (BET) surface area analysis (Figure S7) further elucidates the basis for this superior performance: compared with DFO, GTDHEFO possesses a larger specific surface area (13.04 m^2^ g^−1^) and a higher mesopore volume (0.087 cm^3^ g^−1^). This optimized textural structure not only maximizes the exposure of surface active sites, thereby improving sensitivity, but also significantly reduces gas‐phase Knudsen diffusion resistance, thus accelerating reaction kinetics. Such kinetic advantages of the high‐entropy configuration are fully reflected in the transient response curves (Figure 3a,b). Upon exposure to 3 ppm H_2_S, the GTDHEFO sensor achieves a response time (*t*_res_) of 7.09 s and an ultra‐fast recovery time (*t*_rec_) of 1.72 s. Although its initial response is slightly slower than that of DFO (*t*_res_ = 5.32 s), the GTDHEFO sensor recovers almost instantaneously to the baseline (*t*_rec_ = 1.72 vs. 3.93 s). This indicates a highly reversible surface reaction and a significantly lowered desorption energy barrier. In addition to extremely high sensitivity and excellent kinetic characteristics, outstanding selectivity remains a key factor to ensure practical application. Figure 3c presents the response values of GTDHEFO and DFO to a series of interfering gases at a concentration of 500 ppm. These interfering gases include typical VOCs and industrially relevant gases, such as NH_3_, SO_2_, NO_2_, HCl, trimethylamine, acetonitrile, benzaldehyde, ethylenediamine, and triethylamine. The complete list of tested gases and their corresponding response values is summarized in Table S5. Only a few gases, such as trimethylamine, HCl, and acetonitrile, produced measurable responses, but their response values remained much lower than that to H_2_S. These results demonstrate the excellent selectivity and anti‐interference capability of GTDHEFO for trace H_2_S detection.

Figure 3d further illustrates the dynamic sensing performance of the sensor, demonstrating its capability to track stepwise changes in H_2_S concentration from 1 to 5 ppm in real time. An excellent linear relationship is observed between the response value and the concentration (Figure 3e), with the fitted equation expressed as *y* = 6.120*x* + 0.108 (*R* ^2^ = 0.996). This high sensitivity, together with the outstanding linearity, demonstrates the sensor's tremendous potential for quantitative detection of trace H_2_S at low‐ppm levels. The reliability of the sensor is then rigorously evaluated. Figure 3g presents twelve consecutive response‐recovery cycles, confirming excellent reproducibility. Statistical analysis of these cycles (Figure 3f) reveals a narrow distribution of kinetic parameters. Interestingly, a slight reduction in the response and recovery times is observed after the fifth cycle, which may be attributed to gradual activation of surface active sites and the subsequent attainment of a dynamic equilibrium in the surface adsorption‐desorption process as a result of repeated gas exposure. The humidity‐dependent sensing behavior was further evaluated to assess the environmental adaptability of GTDHEFO (Figure 3h and Figure S8). The sensor maintains stable response‐recovery behavior toward 3 ppm H_2_S under different RH conditions, and the response increases progressively as the RH increases from 30% to 90%. This humidity‐assisted enhancement may be associated with water‐assisted proton/ionic conduction and surface‐coordination‐mediated charge transfer at exposed RE^3+^/Fe^3+^ Lewis acid sites, which can facilitate H_2_S dissociation and interfacial charge transport [45, 46, 47, 48]. Long‐term stability tests (Figure 3i) demonstrate that the sensor retains approximately 90% of its initial response after 30 days of cyclic testing, underscoring the outstanding stability of the high‐entropy lattice in resisting chemical degradation. The operating‐temperature dependence of the GTDHEFO sensor was evaluated toward 5 ppm H_2_S from 298 to 338 K (Figure S9a). Although the baseline current increases with temperature (Figure S9b), the response decreases progressively and reaches its maximum value of approximately 19.20 at 298 K.

**FIGURE 4:**
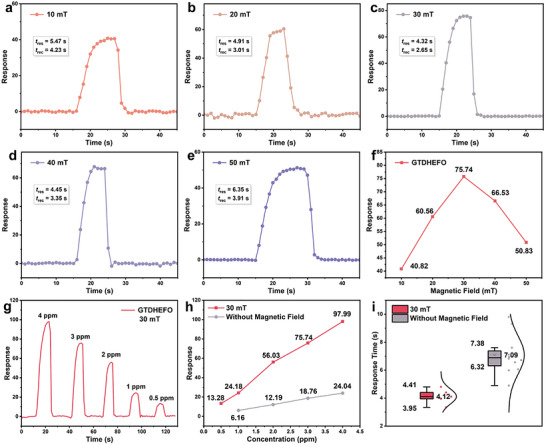
Magnetic field‐modulated sensing performance. (a–e) Transient response‐recovery curves of the GTDHEFO sensor to 3 ppm H_2_S under magnetic fields of 10, 20, 30, 40, and 50 mT, respectively. (f) The “volcano‐shaped” dependence of the response value on magnetic field intensity, peaking at 30 mT. (g) Dynamic response curves to stepwise H_2_S concentrations (0.5–4 ppm) at 30 mT, demonstrating a lowered detection limit. (h) Comparative linearity plots of response vs. concentration for 0 and 30 mT conditions. (i) Statistical box plots of response times collected from multiple cycles, showing a ∼39% reduction in mean response time under the 30 mT field compared to the zero‐field state.

To overcome the sensitivity limit of the sensor, the modulation effect of an external magnetic field on the gas‐sensing performance was investigated. Figure 4a–e present the transient response‐recovery curves of the GTDHEFO sensor toward 3 ppm H_2_S under various static magnetic flux densities ranging from 10 to 50 mT. A significant enhancement in the sensor signal is observed upon application of the magnetic field. As summarized in Figure 4f, the response value exhibits a typical “volcano‐shaped” dependence on the magnetic flux density: it rises sharply from 40.82 at 10 mT to a peak value of 75.74 at 30 mT, and then declines to 50.83 at 50 mT. Notably, the optimal response at 30 mT is approximately four times that under the magnetic‐field‐free condition (18.76), confirming that the magnetic field can serve as an effective external stimulus to modulate the sensing performance. This magnetic‐field‐dependent modulation is also reflected in the dynamic current–time (*I*–*t*) curves (Figure S10), where the response current increases from 0 to 30 mT and then decreases at 50 mT during H_2_S exposure, consistent with the trend in Figure 4f. In addition to sensitivity, the magnetic field also significantly lowers the limit of detection (LOD). Under the optimized magnetic flux density of 30 mT, the sensor is capable of distinguishing H_2_S concentrations as low as 0.5 ppm (Figure 4g), which is lower than the detection limit of 1 ppm in the absence of a magnetic field. A comparative linear analysis (Figure 4h) reveals that the sensitivity slope increases substantially from the magnetic‐field‐free condition to the 30 mT condition, indicating a superior signal‐to‐noise ratio at trace levels. This performance enhancement under the magnetic field is attributed to the synergistic amplification of the high‐entropy‐induced active‐lattice distortion and the magnetic‐field‐regulated charge‐transport process. Furthermore, an activated‐sludge incubation experiment was conducted to evaluate the practical applicability of magnetic‐field‐assisted H_2_S sensing in a simulated biological environment (Figure S11). Activated sludge and anaerobic digestion systems are representative environments involving sulfate‐reduction‐related H_2_S generation [49, 50, 51]. In the N_2_‐purged sealed system containing 5 g of activated sludge culture at pH = 6, the sensor operated under 30 mT exhibits a rapid response increase followed by a plateau after approximately 4 h, demonstrating its capability for continuous tracking of H_2_S‐related signals in a complex anaerobic system.

Figure 4i quantitatively presents the statistical analysis of the kinetic advantage. The distribution of *t*_res_ across multiple sensing cycles confirms a significant acceleration in electron transport kinetics. The average response time is reduced from 7.09 s under 0 mT to 4.32 s under 30 mT, representing a reduction of approximately 39%. This kinetic acceleration under 30 mT, where the response and recovery times are optimized to 4.32 and 2.65 s, respectively (Figure 4c), indicates that the magnetic field not only amplifies the signal but also fundamentally alters the rate‐determining step of the surface reaction. The physical mechanism underlying this magnetosensitive sensing enhancement can be attributed to the synergistic action of the “spin valve” effect and the “port‐docking” effect [52]. First, the accelerated kinetics originate from the spin valve effect. The rare‐earth ferrite possesses a complex canted antiferromagnetic structure exhibiting weak ferromagnetism. An externally applied magnetic field effectively modulates the magnetic domain orientation on the material surface, transforming it from a disordered to an ordered state. This spin ordering substantially reduces the spin scattering barrier at grain boundaries and interfaces, effectively opening a valve for electron transport. Consequently, when electrons are injected by H_2_S, they are transported with lower energy dissipation and at higher speeds, thereby achieving an ultrafast response. Second, the ultrahigh sensitivity and low detection limit arise from the port‐docking effect. The 30 mT magnetic field exploits the paramagnetism of oxygen molecules [26] and the weak ferromagnetism of the material surface [5, 24, 25] to generate a strong local magnetic gradient. This gradient constructs a physisorption capture zone that enriches oxygen species near the surface without altering the static chemical baseline. This magnetically induced concentration effect greatly increases the probability of effective collisions between H_2_S molecules and surface active sites (i.e., port docking). As a result, a strong surface reaction signal can be triggered even at extremely low concentrations (0.5 ppm), significantly enhancing the overall response magnitude. The pronounced manifestation of the spin valve and port‐docking effects is predicated upon the intrinsic properties of the high‐entropy perovskite. The substantial lattice microstrain and abundant local spin‐charge coupling interfaces arising from multi‐principal‐element occupancy provide a high density of active sites and a tunable electronic environment for magnetic field modulation. Therefore, the performance enhancement under a magnetic field is not an isolated external field effect but rather a precise amplification of the inherent advantages of the high‐entropy material. This fully demonstrates the substantial potential of cross‐scale synergistic engineering in overcoming sensing bottlenecks.

**FIGURE 5:**
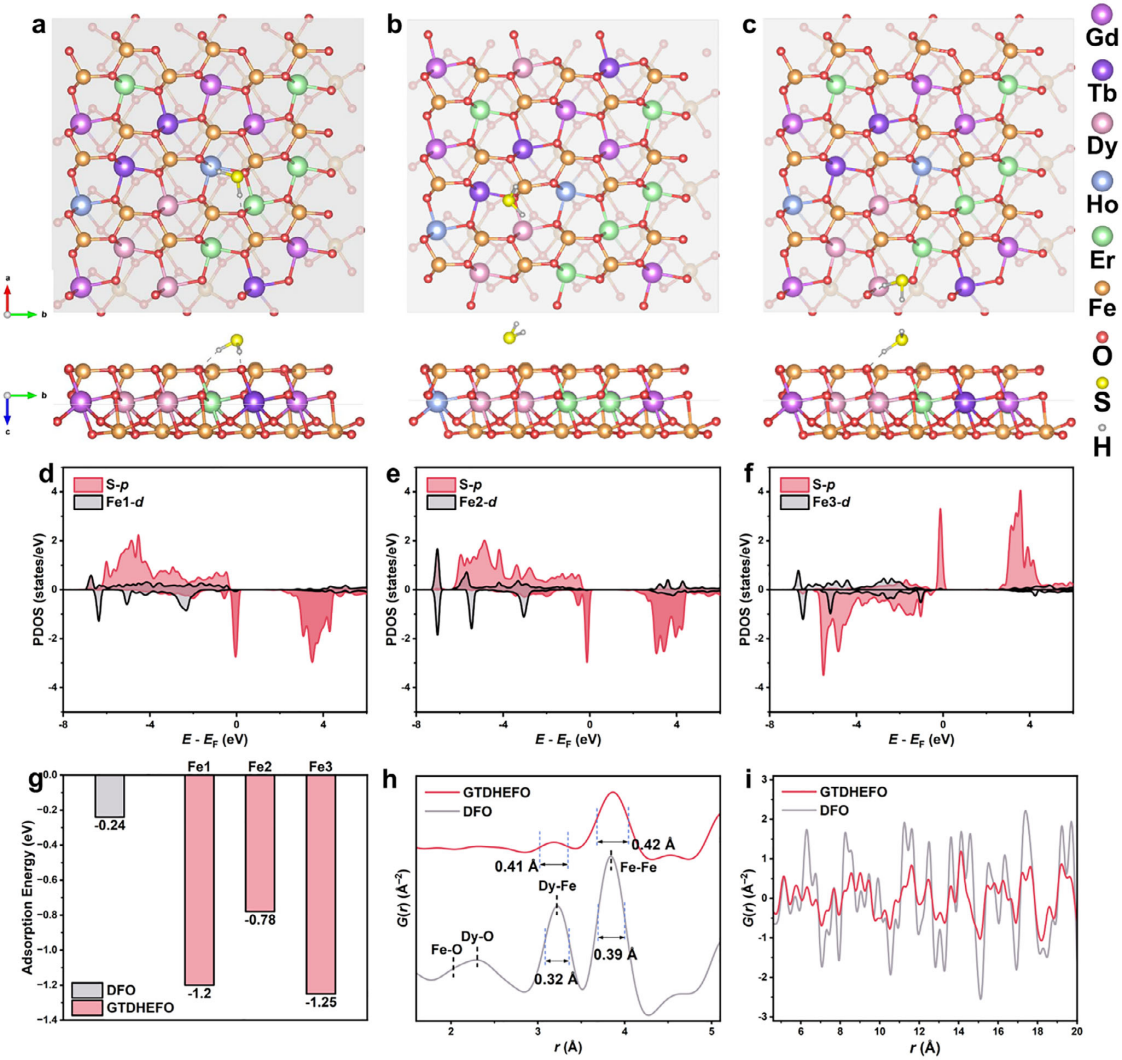
Atomic‐scale structural distortion and electronic mechanisms underlying the high‐entropy effect. (a–c) DFT‐optimized adsorption models of an H_2_S molecule on three distinct Fe active sites (Fe1, Fe2, and Fe3) upon the GTDHEFO (010) surface. (d–f) Corresponding PDOS for the S 3*p* and Fe 3*d* orbitals at the three localized sites. (g) Comparison of calculated H_2_S adsorption energies between the uniform site of DFO and the diverse active sites of GTDHEFO. (h) Experimental Pair Distribution Function, *G*(*r*), for GTDHEFO and DFO in the short‐range region (*r* < 5 Å), with key atomic pairs assigned. (i) Extended range *G*(*r*) profiles up to 20 Å illustrating the characteristic amplitude attenuation induced by severe local lattice distortion.

Subsequently, DFT modeling was performed on the FeO_2_‐terminated (010) surface to evaluate the adsorption energy (*E*_ads_) of H_2_S at the active Fe sites. In contrast to the uniform Fe sites in DFO (Figure S12), the “cocktail effect” within the high‐entropy lattice generates a highly heterogeneous coordination environment. Accordingly, three representative Fe sites, designated Fe1, Fe2, and Fe3, were constructed in GTDHEFO, with each site surrounded by rare‐earth atoms of varying types and at different distances (Figure 5a–c). The calculated adsorption energy of H_2_S on the DFO(010) surface (Figure 5g) is only −0.24 eV, indicating a relatively weak physical interaction. In stark contrast, the diverse active sites on GTDHEFO yield markedly enhanced adsorption energies, with values of −1.20, −0.78, and −1.25 eV for Fe1, Fe2, and Fe3, respectively. To further evaluate the selectivity mechanism, adsorption energy calculations were also performed for representative interfering gas molecules, including H_2_O_2_, CH_3_OH, and (CH_3_)_3_N (Figure S13). The calculated adsorption energies are −0.42, −0.57, and −0.52 eV, respectively, which are less negative than those of H_2_S on the representative GTDHEFO sites. This comparison indicates that H_2_S interacts more strongly with the heterogeneous Fe active sites of GTDHEFO, which is favorable for selective H_2_S adsorption and interfacial charge transfer. Projected density of states (PDOS) analysis further corroborates the electronic origin of this enhanced chemisorption. As shown in Figure 5d–f, the PDOS peaks of the S 3*p* orbitals in the adsorbed H_2_S molecule strongly overlap with those of the 3*d* orbitals of the surface Fe atoms over the energy range from approximately −6.0 to −0.5 eV relative to the Fermi level. This extensive energy overlap indicates strong orbital hybridization between the Fe 3*d* and S 3*p* states (see Figure S14 for comparative data on DFO). Such hybridization supports the formation of Fe–S bonding interactions at these diverse active sites, thereby facilitating H_2_S chemisorption and subsequent interfacial charge transfer, which ultimately contributes to the improved macroscopic gas‐sensing performance.

To further clarify the conductivity type of GTDHEFO and the origin of the current response, Mott‐Schottky measurements were performed (Figure S15). The plots recorded at 1000, 1500, and 2000 Hz exhibit positive slopes in the linear regions, confirming the *n*‐type semiconducting behavior of GTDHEFO. Linear extrapolation to (*C* ^−2^ = 0) gives a flat‐band potential of approximately −1.02 V vs. Ag/AgCl. This n‐type character is consistent with the positive current response: exposure to the reducing gas H_2_S increases the current relative to that in air and decreases the resistance.

Finally, synchrotron X‐ray PDF analysis was performed to elucidate the local structural origin of the enhanced H_2_S sensing performance. As shown in Figure 5h,i, the peak positions and oscillatory phases of the *G*(*r*) profiles of DFO and GTDHEFO remain essentially unchanged, indicating that the average perovskite framework is preserved after high‐entropy engineering. In contrast, GTDHEFO exhibits pronounced attenuation of the PDF amplitudes and broadening of the characteristic atomic‐pair peaks. The full widths at half maximum of the Fe─*R* and Fe─Fe peaks increase from 0.32 and 0.39 Å in DFO to 0.41 and 0.42 Å in GTDHEFO, respectively. The reduced amplitudes primarily reflect a broader distribution of atomic‐pair distances induced by configurational disorder rather than a decrease in coordination number. Random occupation of the A‐site by Gd, Tb, Dy, Ho, and Er generates heterogeneous local strain fields, broadening the *R*─O, Fe─*R*, and Fe─Fe distance distributions and consequently reducing the peak intensities. As these local distortions accumulate over longer distances, the intermediate‐range structural coherence is progressively weakened, resulting in stronger damping of the *G*(*r*) oscillations. Furthermore, the characteristic PDF features remain stable when different *Q*_max_ values are used for data processing (Figure S16), excluding Fourier truncation as the primary origin of the attenuation. Together with the comparable particle sizes and morphologies of DFO and GTDHEFO, these results indicate that the attenuated *G*(*r*) profile of GTDHEFO mainly originates from configurational‐disorder‐induced local lattice distortion, while the average orthorhombic perovskite framework remains intact.

## 3 Conclusion

In summary, this study reports a novel strategy for modulating local symmetry breaking in perovskite oxides through configurational entropy engineering. A multi‐principal‐element A‐site sublattice was precisely constructed using the sol–gel method, with GTDHEFO as the model system. The results demonstrated that the high‐entropy effect induces an intrinsic lattice microstrain of up to 0.12% in GTDHEFO. This microstrain originates from a significant reduction in the effective coordination number at the A‐site and an increased cooperative tilt angle of FeO_6_ octahedra at the atomic scale. In terms of sensing performance, the local distortion within GTDHEFO significantly enhances surface activity, leading to a 6‐fold increase in the H_2_S response value. By optimizing the adsorption energy barrier through diverse active sites, the system achieved exceptionally high reversibility of desorption and reduced the recovery time to 1.72 s. Notably, a weak magnetic field of 30 mT effectively modulates the spin ordering, producing “spin valve” and “port‐docking” effects, which further improve the response value by a factor of four. DFT orbital analysis confirms that the heterogeneous coordination environment induced by the high‐entropy configuration strengthens the orbital hybridization between Fe 3*d* and S 3*p* orbitals, thereby substantially lowering the interfacial charge transfer barrier. Overall, the inherent “cocktail effect” in the high‐entropy lattice creates heterogeneous active sites unattainable in conventional materials, providing theoretical support for overcoming the response‐recovery trade‐off in semiconductor gas sensing applications. Furthermore, the introduction of a magnetic‐field‐assisted enhancement mechanism points toward a new direction for the design and development of multimodal high‐entropy intelligent sensing systems exhibiting magneto‐electronic characteristics.

## 4 Experimental Section

### 4.1 Material Synthesis

The perovskite oxides (GdFeO_3_, TbFeO_3_, DyFeO_3_, HoFeO_3_, and ErFeO_3_) and the high‐entropy perovskite oxide (Gd_0.2_Tb_0.2_Dy_0.2_Ho_0.2_Er_0.2_)FeO_3_ were synthesized via a facile sol‐gel method. Specifically, stoichiometric amounts of analytical‐grade metal nitrate precursors—Gd(NO_3_)_3_·6H_2_O (99.90%), Tb(NO_3_)_3_·6H_2_O (99.90%), Dy(NO_3_)_3_·6H_2_O (99.99%), Ho(NO_3_)_3_·5H_2_O (99.90%), Er(NO_3_)_3_·5H_2_O (99.90%), and Fe(NO_3_)_3_·9H_2_O (99.70%), all purchased from Aladdin, China—were dissolved in deionized water. Subsequently, citric acid was added as a chelating agent with a 2:1 molar ratio of citric acid to total metal ions. The mixture was continuously stirred until a clear solution was formed. The solution was then heated in a thermostatic magnetic stirring water bath at 353 K for 10 h under magnetic stirring to promote gelation. The resulting wet gel was dried in a forced‐air oven for 36 h to obtain the xerogel. Finally, the dried precursor powder was transferred into a corundum crucible and annealed at 973 K for 2.5 h in air to yield the highly crystallized nanoscale perovskite oxide samples.

### 4.2 Material Characterization

The elemental composition was determined via inductively coupled plasma optical emission spectrometry (ICP‐OES, Agilent 5110). Phase and crystal structure were identified by X‐ray diffraction (XRD, Bruker D8 Advance, Cu K*α* radiation) with Rietveld refinement performed using TOPAS software. Microstructural morphology and elemental distribution were characterized using field‐emission scanning electron microscopy (FE‐SEM, Zeiss Supra 55 VP) and transmission electron microscopy (TEM, FEI Talos 200X, 200 kV) equipped with a SuperX EDS detector. Room‐temperature Raman spectra were acquired on a LabRAM HR Evolution spectrometer (532 nm laser). Surface chemical states were analyzed by X‐ray photoelectron spectroscopy (XPS, Thermo Fisher Scientific K‐Alpha, Al K*α* source), and all binding energies were calibrated to the adventitious C 1*s* peak at 284.8 eV. Electron paramagnetic resonance (EPR) measurements were conducted on a Bruker ELEXSYS‐II E500 spectrometer to investigate defect states. O_2_‐TPD and O_2_‐H_2_‐TPD‐MS were conducted on a Micromeritics AutoChem 2950 HP instrument. The sample was pretreated under He at 673 K, cooled to 323 K, exposed to 10% O_2_/He for 1 h, and then purged with He for 1 h. Subsequently, the sample was heated to 973 K at 10 K min^−1^ under He, and the desorbed species were detected by TCD and/or MS. Only the signals collected in the 323–523 K range were used for analysis. Nitrogen adsorption‐desorption isotherms (Micromeritics ASAP 2460) were used to calculate the specific surface area via the Brunauer‐Emmett‐Teller (BET) method. Mott‐Schottky measurements were performed on an electrochemical workstation (CHI‐660E) using Ag/AgCl as the reference electrode under an air atmosphere at frequencies of 1000, 1500, and 2000 Hz. To probe the local structural evolution in detail, high‐resolution synchrotron X‐ray total scattering (ESRF, beamline ID31; 75.051 keV, *λ* = 0.16520 Å) was utilized. The pair distribution function (PDF) was extracted through conventional data reduction with a Lorch correction, utilizing a high *Q*_max_ to achieve superior real‐space resolution [53, 54, 55].

### 4.3 Fabrication and Measurement of the Gas Sensor

To fabricate the gas sensing devices, a specified amount of the prepared perovskite powder was mixed with a few drops of deionized water and ground in an agate mortar to form a homogeneous paste. This paste was then drop‐coated onto the surface of pre‐cleaned Ag‐Pd interdigitated electrodes (13.4 mm × 7 mm) and allowed to dry naturally at room temperature for 24 h.

Gas sensing performance, particularly toward target gases such as H_2_S, was evaluated utilizing a commercial photoelectric integrated testing platform (CGS‐MT, China). Specific target gas concentrations were generated via a static volumetric dilution method (Note: Liquid VOC interferents were vaporized using a constant temperature evaporation method). The sensor response is defined as the normalized change in current when exposed to the target gas, calculated by the following equation:

(1) $$\mathrm{Response}\;=\frac{\Delta I}{I_{\mathrm{air}}}=\frac{I_{\mathrm{gas}}-I_{\mathrm{air}}}{I_{\mathrm{air}}}\;$$

where *I*_gas_ and *I*_air_ represent the measured current of the sensor in the target gas and in ambient air, respectively. The response and recovery time, serving as critical dynamic indicators, are defined as the time required for the sensor's output signal to reach 90% of its stable value upon exposure to the target gas and to recover by 90% of the maximum signal change upon removal of the gas, respectively.

To evaluate the sensor under a simulated biological environment, 5 g of activated sludge culture with pH = 6 was placed in a sealed glass bottle. The bottle was purged with N_2_ to create an anaerobic atmosphere. The GTDHEFO sensor was positioned in the headspace and operated at room temperature under a static magnetic flux density of 30 mT to monitor the gas generated from the activated‐sludge system.

To investigate the magnetic‐field‐modulated gas‐sensing behavior, a one‐dimensional Helmholtz coil system (LM2100‐090‐050, China) was integrated into the gas‐sensing test chamber (Figure S17). The external magnetic field used in this study was a static magnetic field generated under DC excitation rather than an alternating magnetic field. When driven by a DC excitation power supply, the Helmholtz coil pair generated a spatially uniform magnetic‐field region between the two coils, with the magnetic‐field direction parallel to the coil axis. The gas sensor was positioned at the center of this uniform‐field region using a rotating/lifting stage. The magnetic flux density *B*, was controlled by adjusting the excitation current and followed the linear relationship (*B*  =  *kI*), where *k* is the coil constant and *I* is the excitation current. The magnetic flux density could be continuously adjusted from 0 to 53.29 mT along the positive axial direction of the coil. Magnetic‐field‐dependent sensing measurements were performed at 0, 10, 20, 30, 40, and 50 mT.

### 4.4 DFT Calculation Details

All spin‐polarized DFT calculations were performed using the Vienna Ab initio Simulation Package (VASP) based on the projector augmented‐wave (PAW) method [56, 57, 58, 59]. The exchange‐correlation interactions were described by the generalized gradient approximation (GGA) with the Perdew‐Burke‐Ernzerhof (PBE) functional [60]. To accurately describe the van der Waals (vdW) interactions, the Grimme DFT‐D3 empirical dispersion correction was incorporated [61]. The plane‐wave cut‐off energy was set to 500 eV. To eliminate spurious periodic interactions, a vacuum layer of 15 Å was applied along the *z*‐direction for all surface models. The Brillouin zone was sampled using a Γ‐centered 1 × 1 × 1 *k*‐point mesh. The energy and force convergence criteria for the structural relaxations were strictly set to 10^−5^ eV and 0.02 eV/Å, respectively. Furthermore, to account for the strong on‐site Coulomb interactions of the localized *d*‐electrons of the Fe atoms, the DFT+U approach [62] was employed with an effective Hubbard value (*U*_eff_ =  *U* − *J*) of 4.0 eV.

The adsorption energy (Δ*E*_ads_) of the H_2_S molecule on the surface was evaluated using the following equation:

(2) $$\Delta E_{\mathrm{ads}}=E_{\mathrm{total}}\;-E_{\mathrm{sur}}-E_{H2S}$$

where *E*_total_, *E*_sur_, and *E*_H2S_ represent the total energies of the adsorption system, the clean surface, and the isolated H_2_S molecule, respectively.

## Author Contributions

**Yunfei Wang**: writing – original draft, data curation, methodology, visualization, conceptualization, investigation. **Zhaofeng Wu**: validation. **Rui Li**: validation, supervision. **Zhenjiang Li**: formal analysis. **Shan Qiu**: data curation. **Xiaolong Yao**: software. **Min Zhang**: writing – review and editing, project administration, funding acquisition, validation, resources. **Fengdong Qu**: validation.

## Conflicts of Interest

The authors declare no conflicts of interest.

## Supporting information




**Supporting File**: advs76945‐sup‐0001‐SuppMat.docx.

## Data Availability

The data that support the findings of this study are available from the corresponding author upon reasonable request.
