# Supplementary material for: High‐Entropy Engineering and External Magnetic Field Modulation Synergistically Enhance the H2S Sensing Performance of Rare‐Earth Perovskite Ferrites
Source: Adv Sci (Weinh). 2026 Aug 5:e76945. Online ahead of print. doi: 10.1002/advs.76945 (PMC13440365; doi:10.1002/advs.76945)
Supplement: Supplementary file 1 [file ADVS-9999-e76945-s001.docx]

Supporting Information

High-Entropy Engineering and External Magnetic Field Modulation Synergistically Enhance the H_2_S Sensing Performance of Rare-Earth Perovskite Ferrites

Yunfei Wang, Min Zhang*, Rui Li*, Xiaolong Yao, Zhaofeng Wu, Fengdong Qu*, Shan Qiu, Zhenjiang Li

**Text S1. Calculation of Tolerance Factor (*t*)**

In the ABO_3_ perovskite systems, the Goldschmidt tolerance factor (*t*) serves as a quantitative metric to evaluate both the stability of the ideal perovskite lattice and the degree of structural distortion. It is defined as:

$$t=\frac{R_{A}+R_{O}}{\sqrt{2}(R_{B}+R_{O})}$$

(S1)

where $R_{A}$, $R_{B}$, and $R_{O}$ represent the effective ionic radii of the A-site, B-site, and O-site ions, respectively. For the multi-component A-site, $R_{A}$ was calculated using a compositionally weighted average. Assuming an ideal perovskite framework, the coordination numbers (C.N.) are assigned as 12 for the A-site, and 6 for both the B-site and O-site. All effective ionic radii were sourced from Shannon (1976). Because Shannon's database lacks C.N. = 12 radii for certain rare-earth ions, these values were extrapolated using Zachariasen’s^[1]^ empirical relationship between ionic radius and coordination number, as tabulated by Jia^[2]^.

Because these calculated tolerance factors for all synthesized structures fall within the narrow interval of 0.90 to 0.93, the crystal lattice is anticipated to deviate from ideal cubic symmetry, adopting an orthorhombic or rhombohedral structure primarily driven by the cooperative tilting of the BO_6_ octahedra.

| **Sample** | **GFO** | **TFO** | **DFO** | **HFO** | **EFO** | **GTDHEFO** |
| --- | --- | --- | --- | --- | --- | --- |
| ***t*** | 0.9234 | 0.9164 | 0.9130 | 0.9095 | 0.9061 | 0.9137 |

**Text S2. Calculation of Lattice Microstrain (*ε*)**

The Williamson–Hall model is a method for separating crystallite size and lattice microstrain from X‑ray diffraction (XRD) data. After the instrumental broadening is subtracted, the full width at half maximum (FWHM) of the diffraction peaks obtained from XRD refinement can be employed to quantify the degree of lattice microstrain by means of Equation S2:

$$\beta_{T}=\beta_{D}+\beta_{\varepsilon}=\frac{K\lambda}{D\cdot\cos\theta}+4\varepsilon\cdot\frac{\sin\theta}{\cos\theta}$$

(S2)

$$\beta_{T}\cdot\cos\theta=\frac{K\lambda}{D}+4\varepsilon\cdot\sin\theta$$

(S3)

Where, $\beta_{T}$ is the full width at half maximum, $\beta_{D}$ is the broadening arising from crystallite size, and $\beta_{\varepsilon}$ is the broadening induced by strain; $K$ = 0.9 is the shape factor, $\lambda$ is the wavelength of the X‑ray source, $D$ is the crystallite size, and $\theta$ is the peak position expressed in radians. Equation S3, which relates $4\sin\theta$ and $\beta_{T}\cdot\cos\theta$, is derived from Equation S2.

**Text S3. Calculation of Effective Coordination Number (ECoN)**

The degree of polyhedral distortion is investigated by calculating the average ECoN of the A-site and B-site ions with respect to the adjacent O-site ions. ECoN reflects the actual coordination environment through a bond-length-weighted average of the contributions from A-site and B-site atoms, while simultaneously revealing characteristics of short-range order. The ECoN can be obtained using Equation S4 and S5:

$$\mathrm{ECoN}=\sum\exp[1-{(\frac{l_{i}}{l_{\mathrm{av}}})}^{6}]$$

(S4)

$$l_{\mathrm{av}}=\frac{\sum l_{i}\cdot exp[1-{({l_{i}}/{l_{\min})}}^{6}]}{\sum exp[1-{({l_{i}}/{l_{\min})}}^{6}]}$$

(S5)

Where $l_{i}$ denotes the bond length between the A-site or B-site ion and the O-site ion obtained via Rietveld refinements, $l_{\mathrm{av}}$ denotes the bond length determined by the weighted average method, and $l_{\min}$ represents the shortest bond length between the A-site or B-site ion and the O-site ion.

**Text S4. Calculation of Lattice Strain (***ϵ***)**

In orthorhombic structures characterized by low-symmetry distortion, minor discrepancies in axial lengths arise from factors such as octahedral tilting and ionic substitution; nevertheless, the overall lattice retains a nearly cubic character, and the unit cell exhibits an approximate cubic symmetry in macroscopic diffraction patterns (Equation S6). To eliminate dimensional disparities, the multi-axial and anisotropic unit cell dimensions are transformed, through mathematically rigorous conversions consistent with crystallographic principles, into a single, isotropic equivalent cubic lattice constant (Equation S7 and S8). The three normalized values derived via the equal-volume equivalence approach are subsequently employed in Equation S9 to determine the degree of lattice distortion relative to the ideal cubic phase.

$$a_{ps.cubic}={V_{f.u.}}^{\frac{1}{3}}={(\frac{V}{4})}^{\frac{1}{3}}$$

(S6)

$${(a, c)}_{\mathrm{norm}}=\frac{a,c}{\sqrt{2}{\times a}_{ps.cubic}}$$

(S7)

$$b_{\mathrm{norm}}=\frac{b}{2{\times a}_{ps.cubic}}$$

(S8)

$$\epsilon=\frac{1}{3}\times{(a_{\mathrm{norm}}+b_{\mathrm{norm}}+c_{\mathrm{norm}})}^{\frac{1}{2}}$$

(S9)

where $a$, $b$, $c$ and $V$ denote the lattice parameters refined via Rietveld refinement, $a_{ps.cubic}$ and $V_{f.u.}$ denote the pseudo-cubic lattice parameters, respectively. The notation ${(a,b, c)}_{\mathrm{norm}}$ refers to the normalized equivalent cubic lattice constants, and *ϵ* corresponds to the degree of lattice distortion relative to the ideal cubic phase.

**Figure S1.** Rietveld refinements of the XRD patterns of all samples, showing the observed (dots), calculated, and difference (grey line) profiles, along with Bragg positions (vertical bars).

**Figure S2.** Particle size distribution of (a) DFO and (b) GTDHEFO based on microscopy images.

**Figure S3.** Batch-to-batch reproducibility of GTDHEFO synthesized using the same preparation procedure. (a) XRD patterns of three independently synthesized GTDHEFO batches. (b–d) SEM images of Sample 3, Sample 2, and Sample 1, respectively. (e–g) Corresponding particle size distribution analyses of Sample 3, Sample 2, and Sample 1. The average particle sizes are 81.66, 73.88, and 70.60 nm for Sample 3, Sample 2, and Sample 1, respectively.

**Figure S4.** High-resolution XPS spectrum of GTDHEFO in the 130–180 eV region with deconvoluted rare-earth 4*d*-related components assigned to Gd 4*d*, Tb 4*d*, Dy 4*d*, Ho 4*d*, and Er 4*d*. Because of the close binding energies and severe overlap of rare-earth 4*d* signals, the peak fitting is used for qualitative elemental identification rather than quantitative composition analysis.

**Figure S5.** The EPR spectra of DFO and GTDHEFO.

**Figure S6.** Surface oxygen adsorption and reduction reactivity of DFO and GTDHEFO. (a) O_2_-TPD profiles of DFO and GTDHEFO. DFO exhibits a low-temperature O_2_ desorption peak at 373.9 K with a desorption amount of 0.00359 mmol g^–1^, whereas GTDHEFO shows a stronger O_2_ desorption peak at 361.9 K with a desorption amount of 0.06509 mmol g^–1^. O_2_-H_2_-TPD-MS profiles of DFO and GTDHEFO monitored at (b) *m*/*z* = 18, (c) *m*/*z* = 44, and (d) *m*/*z* = 16.

**Figure S7.** N_2_ adsorption and desorption isotherms and their corresponding BJH pore size distributions for DFO and GTDHEFO.

**Figure S8.** Response of GTDHEFO under different relative humidity conditions.

**Figure S9.** Temperature-dependent sensing characteristics of the GTDHEFO sensor toward 5 ppm H_2_S. (a) Baseline current of GTDHEFO at different operating temperatures from 298 to 338 K. (b) Dynamic response–recovery curves toward 5 ppm H_2_S at different operating temperatures.

**Figure S10.** Dynamic current-time (*I*–*t*) curves of the GTDHEFO sensor toward H_2_S under different static magnetic flux densities of 0, 10, 30, and 50 mT.

**Figure S11.** Time-dependent response of the GTDHEFO sensor during activated-sludge incubation under anaerobic conditions. The test was performed using 5 g of activated sludge culture with pH = 6 in a sealed N_2_-purged bottle under a static magnetic flux density of 30 mT at room temperature.

**Figure S12.** DFT-optimized adsorption models of an H_2_S molecule on three distinct Fe active sites upon the DFO (010) surface.

**Figure S13.** DFT-optimized adsorption configurations and corresponding adsorption energies of representative interfering gas molecules on the GTDHEFO (010) surface: (a) H_2_O_2_, (b) CH_3_OH, and (c) (CH_3_)_3_N. The calculated adsorption energies are −0.42, −0.57, and −0.52 eV for H_2_O_2_, CH_3_OH, and (CH_3_)_3_N, respectively.

**Figure S14.** Partial density of states (PDOS) of S-*p* and Fe-*d* orbitals for the DFO perovskite.

**Figure S15.** Mott-Schottky plots of GTDHEFO measured at 1000, 1500, and 2000 Hz using Ag/AgCl as the reference electrode under an air atmosphere.

**Figure S16.** Detailed views of the *G*(*r*) pair distribution functions for the GTDHEFO sample collected at different *r* ranges.

**Figure S17.** Schematic illustration of the static-magnetic-field-assisted gas-sensing measurement configuration.

**Table S1.** The elemental composition of DFO and GTDHEFO.

| **Sample** | **DFO** | | **GTDHEFO** | | | | | |
| --- | --- | --- | --- | --- | --- | --- | --- | --- |
|  | **Dy** | **Fe** | **Gd** | **Tb** | **Dy** | **Ho** | **Er** | **Fe** |
| **Elemental composition (wt.%)** | 65.05 | 22.15 | 12.38 | 11.77 | 13.05 | 12.44 | 12.64 | 21.72 |
| **Molar mass (*M*/g·mol^–1^)** | 162.50 | 55.845 | 157.25 | 158.93 | 162.50 | 164.93 | 167.26 | 55.845 |
| **Amount of substance (*n*/mol)** | 0.4003 | 0.3966 | 0.0787 | 0.0741 | 0.0803 | 0.0754 | 0.0756 | 0.3890 |
| **A-site to B-site molar ratio** | 1 | 0.99 | 0.20 | 0.19 | 0.21 | 0.19 | 0.20 | 1 |
| **Actual composition** | DyFe_0.99_O_3_ | | (Gd_0.21_Tb_0.19_Dy_0.21_Ho_0.19_Er_0.20_)FeO_3_ | | | | | |

**Table S2.** Lattice parameters and cell volume of the samples.

| **Sample** | **Lattice constants** | | | | **Cell volume** | **Space group** |
| --- | --- | --- | --- | --- | --- | --- |
|  | ***a* (Å)** | ***b* (Å)** | ***c* (Å)** | ***α* = *β* = *γ* (deg)** | ***V* (Å^3^)** |  |
| **GTDHEFO** | 5.60092 | 7.63244 | 5.30797 | 90 | 226.908646 | *Pnma* |
| **EFO** | 5.58711 | 7.59929 | 5.26723 | 90 | 223.636358 | *Pnma* |
| **HFO** | 5.59535 | 7.61308 | 5.28539 | 90 | 225.146395 | *Pnma* |
| **DFO** | 5.59823 | 7.62717 | 5.30677 | 90 | 226.591718 | *Pnma* |
| **TFO** | 5.60066 | 7.64661 | 5.33101 | 90 | 228.306360 | *Pnma* |
| **GFO** | 5.61412 | 7.67404 | 5.35294 | 90 | 230.620822 | *Pnma* |

**Table S3.** A/B-O bond lengths (*l*_i_), average bond lengths (*l*_av_) and effective coordination numbers (ECoN) for DFO and GTDHEFO.

| **Sample** | ***l*_i_ *(*A/B-O) (Å)** | | | | | | ***l*_av_ (Å)** | **ECoN** |
| --- | --- | --- | --- | --- | --- | --- | --- | --- |
| **DFO** | 2.31146 | 2.35144 | 2.35144 | 2.38942 | 2.56183 | 2.56183 | 1.0327 | 7.1664 |
|  | 2.61816 | 2.61816 | 3.08118 | 3.31942 | 3.49613 | 3.49613 |  |  |
|  | 1.96696 | 1.96696 | 1.99058 | 1.99058 | 2.01624 | 2.01624 | 0.6338 | 5.9782 |
| **GTDHEFO** | 2.30222 | 2.33958 | 2.33958 | 2.36021 | 2.57154 | 2.57154 | 1.0273 | 7.0196 |
|  | 2.61567 | 2.61567 | 3.10870 | 3.37430 | 3.49897 | 3.49897 |  |  |
|  | 1.98102 | 1.98102 | 1.99156 | 1.99156 | 2.01489 | 2.01489 | 0.6355 | 5.9896 |

**Table S4.** Structural evolution parameters of DFO and GTDHEFO.

| **Sample** | ***V*_f.u._** | ***a*_ps.cubic_** | ***a*_norm_** | ***b*_norm_** | ***c*_norm_** | *ϵ* |
| --- | --- | --- | --- | --- | --- | --- |
| **DFO** | 56.64793 | 3.84056 | 1.03088 | 0.99298 | 0.97721 | 0.42698 |
| **GTDHEFO** | 56.72716 | 3.84235 | 1.03089 | 0.99320 | 0.97697 | 0.42705 |

**Table S5.** Gas sensing responses of the GTDHEFO sensor to various interfering gases at 500 ppm.

| **Chemical Nature** | **Gas Name** | **Chemical Formula** | **Response** |
| --- | --- | --- | --- |
| Acidic | Phenol | C_6_H_5_OH | Negligible |
|  | Hydrogen peroxide | H_2_O_2_ | Negligible |
|  | Sulfur dioxide | SO_2_ | 1.324 |
|  | Nitrogen dioxide | NO_2_ | Negligible |
|  | Hydrochloric acid | HCl | 2.546 |
| Neutral | N-methyl pyrrolidone | C_5_H_9_ON | Negligible |
|  | Dimethyl sulfoxide | C_2_H_6_OS | 0.212 |
|  | 1,4-dioxane | C_4_H_8_O_2_ | Negligible |
|  | Methanol | CH_3_OH | 0.475 |
|  | Formaldehyde | HCHO | Negligible |
|  | Acetone | C_3_H_6_O | Negligible |
|  | Ethylene glycol | C_2_H_6_O_2_ | Negligible |
|  | Ethanol | C_2_H_5_OH | Negligible |
|  | Toluene | C_6_H_5_CH_3_ | Negligible |
|  | N,N-dimethylformamide | C_3_H_7_ON | 0.179 |
|  | Benzaldehyde | C_6_H_5_CHO | Negligible |
|  | Acetonitrile | CH_3_CN | 2.088 |
| Alkaline | Hydrazine | N_2_H_4_ | 0.620 |
|  | Ammonia | NH_3_ | Negligible |
|  | Trimethylamine | (CH_3_)_3_N | 2.017 |
|  | Aniline | C_6_H_5_NH_2_ | 0.339 |
|  | Ethylene diamine | C_2_H_8_N_2_ | Negligible |
|  | Triethylamine | (C_2_H_5_)_3_N | Negligible |

**References**

1.	W.H. Zachariasen, “Bond lengths in oxygen and halogen compounds of d and f elements,” *J. Common Met.* 62 (1978): 1–7, https://doi.org/10.1016/0022-5088(78)90010-3.

2.	Y.Q. Jia, “Crystal radii and effective ionic radii of the rare earth ions,” *J. Solid State Chem.* 95 (1991): 184–187, https://doi.org/10.1016/0022-4596(91)90388-X.
